# Convection Effect of Plasma Flow on Oxygen Transport in Capillaries: An In‐Depth Numerical Investigation

**DOI:** 10.1111/micc.70011

**Published:** 2025-04-20

**Authors:** Junfeng Zhang

**Affiliations:** ^1^ Bharti School of Engineering and Computer Science Laurentian University Sudbury Ontario Canada

**Keywords:** microcirculation, oxygen transport, Peclet number, plasma circulation, red blood cells

## Abstract

**Objective:**

The convection effect of plasma flow on gas transport in the microcirculation has been a controversial topic in the literature. We aim to clarify this concern via thorough and rigorous analysis of the oxygen release process from red blood cells (RBCs) to the surrounding tissue.

**Methods:**

We develop a comprehensive model that considers the plasma flow, RBC deformation, oxygen transport and oxygen‐hemoglobin reaction kinetics. The boundary integral and lattice Boltzmann methods are employed in the numerical solutions. In particular, the oxygen fluxes due to plasma convection and mass diffusion are separately calculated along the capillary wall for further comparison.

**Results:**

Our results show that the most significant diffusive flux occurs in the narrow gap between the RBC side surface and the capillary wall and the diffusive flux is primarily directed outward, which favors oxygen release into the surrounding tissue. Furthermore, although the axial convective flux is the most profound in magnitude, it contributes little to the overall blood‐to‐tissue oxygen transport in the radial direction. The radial convective flux also has a larger magnitude compared to the diffusive oxygen flux, but is limited to two small areas and to opposite directions. This results in a negligible net effect of the plasma convection compared to the diffusive flux on the overall oxygen transport. This observation is further confirmed by comparing the oxygen distributions and diffusive fluxes from simulations with and without considering the plasma convection flow relative to RBCs. Moreover, we revisit the Peclet number definition and propose that different characteristic length scales should be adopted for oxygen diffusion and convection in capillaries. The revised Peclet number has a value three orders of magnitude lower than that from the classical Peclet number definition.

**Conclusions:**

Our simulation results show that the influence of plasma convection on the overall oxygen transport can be neglected in typical microcirculation situations. This is consistent with the revised Peclet number value, suggesting that the revised Peclet number can better reflect the relative importance of convection and diffusion mechanisms in microvascular gas transport.

## 1 Introduction

Blood plays many critical biological functions in human and other vertebrate bodies, including the transport of oxygen from the lungs to tissue cells throughout the body to maintain a continuous oxygen supply for mitochondrial adenosine triphosphate (ATP) production and other biochemical reactions [1, 2, 3, 4]. Blood is a suspension of cellular particles, including red blood cells (RBCs), white blood cells, and platelets. Among them, RBCs are the most important components in several respects. RBCs account for 45% of the blood volume (i.e., hematocrit). The interior cytoplasm has a high concentration of hemoglobin (Hb), which can reversibly absorb and release oxygen. Approximately 98% of the oxygen in the blood is stored inside RBCs in the form of oxyhemoglobin [1]. The overall oxygen transport in our bodies consists of three parts: the uptake process in the lungs, where the deoxygenated RBCs in the venous blood absorb oxygen from the air through the alveolar wall; the transfer of oxygen with the blood flow through the circulatory network; and the release process in tissues, where oxygen is released from RBCs into surrounding tissues. Both the uptake and release processes take place in capillaries, where individual RBCs serve as sinks or sources at the end of the oxygen transport pathway. Meanwhile, due to their abundance and specific cellular properties, RBCs play the key role in determining blood rheology and flow behaviors in the circulatory network. This, in return, also affects oxygen transport.

In capillaries, the lumen space is divided by deformed RBCs into plasma segments along the vessels. The RBC moves as a solid particle with a uniform velocity and steady shape, resulting in local circulation eddies in the plasma segments between two consecutive RBCs. Researchers have noticed this particular configuration, and tremendous effort has been made to study its influence on gas transport. Hellums [5] modified the classical Krogh model to include discrete fluid segments for the blood in a capillary: one represents the RBC and another for the plasma segment between the RBCs. His results showed that the continuum treatment in the Krogh model significantly underestimated the oxygen transport resistance, as it neglected the discrete nature of RBCs in the capillary and also the low diffusivity of oxygen in the cytoplasm. Plasma flow was not considered in the Hellums study. Meanwhile, Aroesty and Gross [6] simplified the plasma segment as a cylindrical block. They solved the flow and gas concentration fields with artificial boundary conditions on the RBC end surfaces and the capillary wall, and their results showed that the plasma convection effect could be neglected. However, using typical microcirculation values, the Peclet number, a non‐dimensional parameter to characterize the relative strengths of convection and diffusion in mass transfer [7], was found to be on the order of unity, which suggests that plasma convection could play a certain role in oxygen transport and should not be ignored [8, 9]. Groebe and Thews [9], also using the cylindrical model, studied the convective effect of plasma flow with the velocity of RBCs up to 4 mm/s and reported that RBC movement can improve oxygen release. Bos et al. [10] questioned the conclusion of Aroesty and Gross [6]. They considered the same configuration and RBC velocity as in the study by Aroesty and Gross [6]; however, they changed the boundary conditions for oxygen mass transfer on the capillary wall. Their results showed that the convective effect in the space between the RBCs could play a significant role when different boundary conditions were applied.

These studies focused only on the plasma segment and neglected the flow and mass transport in the thin plasma layer between the RBC membrane and the capillary wall, and the deformed RBC shape. More importantly, they applied artificial concentration boundary conditions on the RBC end surfaces and capillary wall without sufficient justification, and the resemblance or relevance of these artificial conditions to the real concentration distributions on these boundary surfaces is open to question. To have a more realistic representation, Whiteley et al. [11] studied the oxygen uptake process in the pulmonary capillary, using two parallel plates for the capillary walls and a circular disk for the RBC. They found that the convective term does not significantly affect oxygen transport and could be neglected. However, the two‐dimensional model and the circular shape of the RBC adopted in this study limit the applicability of its findings. Vadapalli et al. [12] simulated the oxygen release process with parachute‐shaped RBCs and found that flow convection played a minor role in oxygen transport. Meanwhile, Merrikh and Lage [13, 14] studied the carbon monoxide (CO) uptake process in the pulmonary capillaries, where RBCs were modeled as solid particles of parachute shape measured in experiments. Their results showed that plasma convection can significantly improve gas transport efficiency, especially in situations with low hematocrit and high RBC velocity.

Based on the above literature review, the significance of plasma convection to the overall gas transport performance in the microcirculation remains a controversial topic. In this study, we calculate the plasma flow, RBC deformation, and oxygen transport in capillaries and examine the oxygen mass flux distribution around RBCs in detail. The two flux components, the diffusive part due to the oxygen gradient and the convective part due to plasma flow, are compared. Our analysis reveals that the convective flux, although stronger than the diffusive flux in some regions, has a negligible net influence on the overall oxygen transport. We also perform a separate set of calculations with plasma flow relative to RBCs completely ignored, as in previous studies [15, 16, 17] and found that turning off plasma flow has almost no influence on oxygen distribution and transport flux results. Moreover, we revisit the definition of the Peclet number and point out that different characteristic lengths should be adopted for the oxygen diffusion and convection processes in the microcirculation. The revised Peclet number, which has value orders of magnitude smaller than that from the classical definition of the Peclet number, can reflect the relative importance of the convective and diffusive contributions to the oxygen transport more accurately and meaningfully. We hope that this study could shed some light on the controversial topic of plasma convection effects and improve our understanding of the complex processes of gas transport in the microcirculation.

## 2 Model Description, Governing Equations, and Simulation Techniques

**FIGURE 1:**
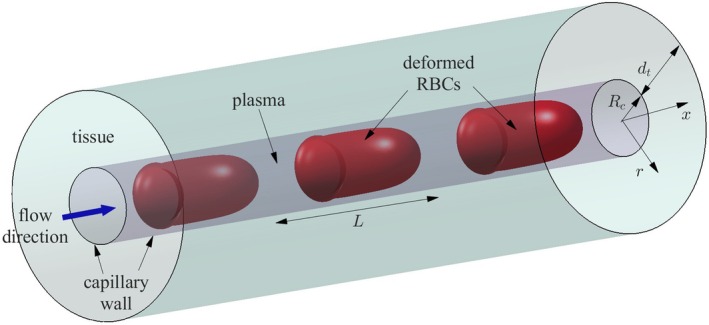
Schematic representation of the axisymmetric system considered in this study. Evenly distributed RBCs flow with ambient plasma through a straight capillary tube of radius $R_{c}$, and the capillary is surrounded by a layer of tissue of thickness $d_{t}$. The separation distance L between RBCs along the capillary is related to the tube hematocrit $H_{t}$.

Following previous studies [16, 18, 19], we adopted an axisymmetric setup as shown in Figure 1, where the deformed RBCs flow along the straight capillary tube at a constant velocity $U_{c}$. The capillary is surrounded by a tissue layer of thickness $d_{t}$. The original RBC has a biconcave shape with a diameter of 7.82 μm, and the corresponding volume and membrane surface area are $V_{\mathrm{RBC}}$ = 94.1 μm^3^ and $A_{\mathrm{RBC}}$ = 134 μm^2^, respectively [20, 21]. The capillary radius is $R_{c}$ = 2.5 μm, and the tube hematocrit is set at $H_{t}$ = 30% [22, 23]. Accordingly, the distance between two RBCs is $L=V_{\mathrm{RBC}}/\pi R_{c}^{2}H_{t}$ = 16.0 μm. The RBC velocity is set as $U_{c}$ = 1 mm/s [24]. The oxygen transport process is described by the following differential equation:

(1) $$\frac{\partial C_{O_{2}}}{\partial t}+u\cdot \nabla C_{O_{2}}=D_{O_{2}}\nabla^{2}C_{O_{2}}+S,$$

where $C_{O_{2}}$ represents the oxygen concentration and is related to the oxygen tension $P_{O_{2}}$ by Henry's law as $C_{O_{2}}=\alpha P_{O_{2}}$ (α is the oxygen solubility). Here $D_{O_{2}}$ is the oxygen diffusivity, and u is the flow velocity. The source term S is related to the oxygen‐hemoglobin reaction rate in the cytoplasm or the oxygen consumption rate in the tissue. S = 0 in the plasma region. This equation is applicable to all domains, including the cytoplasm, plasma, and tissue regions, provided that respective values are used in these domains. When neglecting the interfacial resistance to oxygen transfer across the RBC membrane and capillary wall, the continuity requirements for oxygen concentration $C_{O_{2}}$ and oxygen flux across these surfaces are enforced during the calculation [14, 17, 18, 25].

In addition, in the cytoplasm region, the hemoglobin saturation $S_{O_{2}}$, the fraction of oxyhemoglobin in total hemoglobin, needs to be solved from the following equation:

(2) $$\frac{\partial S_{O_{2}}}{\partial t}+u\cdot \nabla S_{O_{2}}=D_{\mathrm{Hb}}\nabla^{2}S_{O_{2}}-R,$$

where $D_{\mathrm{Hb}}$ is the hemoglobin diffusivity in the cytoplasm? R is the oxygen‐hemoglobin reaction rate, which can be obtained from the Hill function for the equilibrium relationship between $P_{O_{2}}$ and $S_{O_{2}}$

(3) $$S_{O_{2}}=\frac{P_{O_{2}}^{n}}{P_{O_{2}}^{n}+P_{50}^{n}}.$$

In this equation,n is the Hill exponent and $P_{50}$ is the $P_{O_{2}}$ value corresponding to the equilibrium state with $S_{O_{2}}$= 50%. $P_{50}$ is commonly used to characterize oxygen–hemoglobin affinity, and a lower $P_{50}$ value suggests a stronger affinity and vice versa [26]. The reaction rate R in Equation (2) can then be derived from the Hill function as [1, 27]:

(4) $$R=k\left[S_{O_{2}}-\left(1-S_{O_{2}}\right)\frac{P_{O_{2}}^{n}}{P_{50}^{n}}\right],$$

where k is the dissociation rate. The source term S in Equation (1) is related to the reaction rate R by $S=\left[\mathrm{Hb}\right]R$, where $\left[\mathrm{Hb}\right]$ is the hemoglobin concentration in the cytoplasm. The solution of $S_{O_{2}}$ in Equation (2) is limited to the cytoplasmic region, and the no‐penetration boundary condition is applied on the RBC membrane surface [16, 19, 25].

In this study, a tissue layer of $d_{t}$ = 16 μm is adopted, and the oxygen consumption rate $M_{t}$ is considered (i.e., $S=-M_{t}$) in the tissue region [17, 18]. Moreover, the no‐flux condition is applied at the outer tissue boundary $r=R_{c}+d_{t}$ [15, 18, 22]. As for the initial condition, we start with a uniform $P_{O_{2}}$ distribution of 90 mmHg over the entire computational domain. The computational domain has an axial length of L with a single RBC fixed at the center, and periodic boundary conditions applied at the two ends of the tube. Table 1 collects all the simulation parameter values used in this study. The boundary integral method (BIM) is used to obtain the RBC deformation and flow field in the capillary [31, 32, 33], and Equations (1) and (2) are solved using the lattice Boltzmann method [25, 29, 34].

**TABLE 1:** List of property values used in this research.

Property	Value	Reference
Tissue		
$\;\alpha_{t}$	$1.4\times 10^{-9}$ mol/cm^3^ · mmHg	[11]
$\;D_{O_{2},t}$	$2.4\times 10^{-5}$ cm^2^/s	[11]
$\;M_{t}$	$8\times 10^{-8}$ mol/cm^3^ · s	[17]
$\;d_{t}$	16 μm	[17]
Capillary wall		
$\;R_{c}$	2.5 μm	[13, 28]
Plasma		
$\;\alpha_{p}$	$1.4\times 10^{-9}$ mol/cm^3^ · mmHg	[11]
$\;D_{O_{2},p}$	$1.4\times 10^{-9}$ mol/cm^3^ · mmHg	[11]
$\;\mu_{p}$	1.2 cP	[12]
Cytoplasm		
$\;\alpha_{c}$	$1.4\times 10^{-9}$ mol/cm^3^ · mmHg	[11]
$\;D_{O_{2},c}$	$2.4\times 10^{-9}$ cm^2^/s	[11]
$\;D_{\mathrm{Hb}}$	$1.4\times 10^{-7}$ cm^2^/s	[11]
$\;\left[\mathrm{Hb}\right]$	$2.0\times 10^{-5}$ mol/cm^3^	[11]
$\;\mu_{c}$	6.0 cP	[29]
Oxygen‐hemoglobin reaction kinetics		
k	44 1/s	[30]
$\;P_{50}$	26.4 mmHg	[30]
n	2.65	[30]

## 3 Results and Discussion

### 3.1 Plasma Flow Field Around RBCs

**FIGURE 2:**
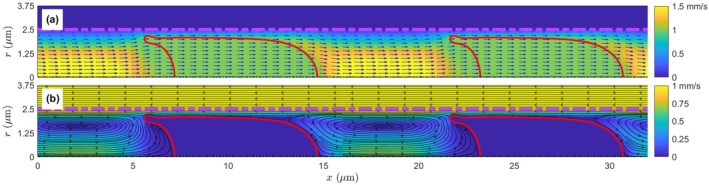
(a) The original and (b) transformed velocity fields utilized in this study. Two simulation units (2 L axial lengths) are displayed here for a better representation of the system configuration and flow structure. For clarity, we use arrows for the velocity vector in (a) and streamlines in (b) to show the velocity directions. In each panel, the deformed RBCs are plotted as solid red curves. The dash‐dotted thick line at r = 2.5 μm indicates the capillary wall location, and the background color scale represents the velocity magnitude over the calculation domain. Only the first 1.25 μm part (r = 2.5–3.75 μm) of the tissue region is shown here for illustration.

While RBCs are moving at $U_{c}$ along the capillary tube in reality, in mathematical analysis and numerical simulations, we usually set the reference frame on a moving RBC, and thus, the RBCs are stationary in the reference frame. This treatment, which has been used in numerous studies [12, 18, 19], avoids the problem of moving boundaries of the RBC membrane and significantly simplifies the numerical solution. Accordingly, the real flow velocity needs to be corrected by subtracting $U_{c}$ for the axial velocity component. Figure 2a shows the flow field in the capillary with the deformed RBCs obtained from the BIM calculation. The cytoplasm velocity is uniform within the RBCs, suggesting that the deformed RBCs are moving as rigid objects with steady velocity and shape. A large shear gradient exists in the gap between the RBC membrane and capillary wall, since the membrane is moving at $U_{c}$ while the capillary is at rest. Away from RBCs (for example, see the segment of x = 16‐21 μm), the plasma velocity exhibits a parabolic‐like profile as the Poiseuille flow: higher near the centerline (∼1.5 mm/s) and gradually reducing to zero near the capillary wall. The transformed velocity in the moving reference frame is shown in Figure 2b for comparison. Now, there is no velocity inside RBCs; however, a constant velocity $U_{c}$ in the reversed axial direction results in the tissue region. The flow circulation in the plasma segment between two RBCs also becomes evident as indicated by the streamlines in Figure 2b. With the circulating eddy, the plasma flows outward behind the rear end of the RBC (x=∼22 μm), and flows inward before the cell front (x =∼ 15 μm); however, the magnitude of such transverse velocity (maximum of 0.163 mm/s) is relatively small compared to the RBC velocity $U_{c}$ (1 mm/s). Similar cell deformation and flow patterns have been reported in the literature [14, 18, 22, 28].

### 3.2 Oxygen Flux Analysis: Diffusive vs. Convective Parts

**FIGURE 3:**
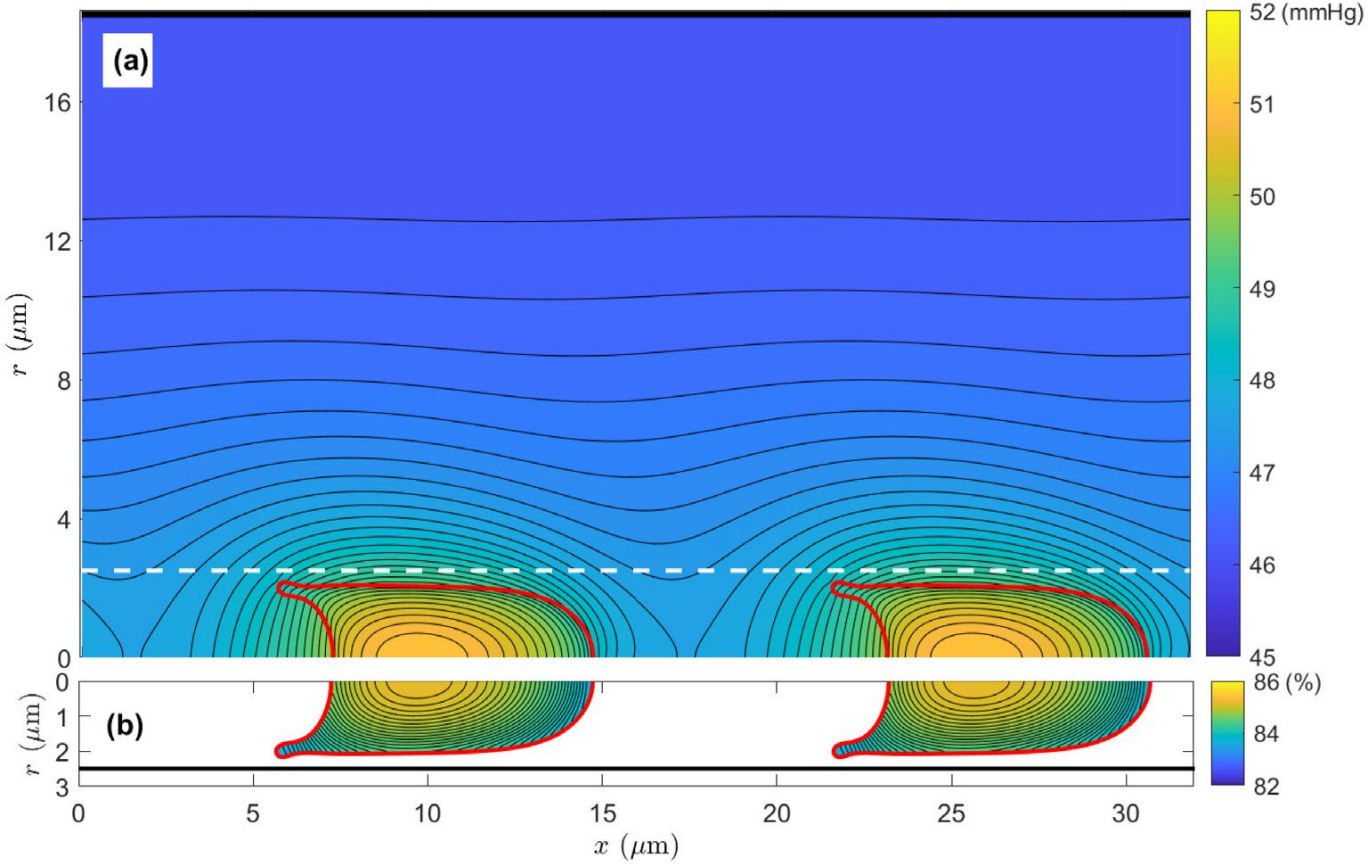
(a) The $P_{O_{2}}$ and (b) $S_{O_{2}}$ distributions at the instant of average cell $P_{O_{2}}$ = 50 mmHg. The white dashed line in (a) and the black solid line in (b) indicate the capillary wall position at r = 2.5 μm, and the deformed RBCs are plotted as red solid curves.

We start the calculation with $P_{O_{2}}$= 90 mmHg in all regions and $S_{O_{2}}$ = 96.3% (the equilibrium saturation at 90 mmHg from the Hill equation Equation 3) in cytoplasm. The calculation result at the early stage should be discarded to exclude the influence of the artificial initial condition [12, 18]. In Figure 3, we present the spatial distributions of $P_{O_{2}}$ and $S_{O_{2}}$ at the instant when the volume‐average $P_{O_{2}}$ in the RBC drops to 50 mmHg. Obviously, the highest $P_{O_{2}}$ is found in RBCs, and it gradually decreases from the RBC membrane in the plasma region inside the capillary tube and the tissue region outside. The $P_{O_{2}}$ and $S_{O_{2}}$ distributions appear asymmetric about the RBC center due to the particular deformed RBC shape and the relative motion of the RBC and plasma fluid to stationary tissues. Inside the RBCs, the $P_{O_{2}}$ and $S_{O_{2}}$ also exhibit some spatial variations, with the maximum values found near the rear end on the centerline. This is reasonable since the cell is moving in the capillary tube to the right, and therefore a lower $P_{O_{2}}$ region results in the front (right) part of the RBC. Similar distributions have been reported in previous studies of biological gas transport [15, 16, 18] and other heat or mass transfer processes [35, 36].

With the $P_{O_{2}}$ distribution in Figure 3a, one can obtain the oxygen diffusive flux through Fick's law:

(5) $$j^{d}=\left(\begin{array}{c} j_{x}^{d}\\ j_{r}^{d} \end{array}\right)=\left(\begin{array}{c} -D_{O_{2}}\frac{\partial C_{O_{2}}}{\partial x}\;\\ -D_{O_{2}}\frac{\partial C_{O_{2}}}{\partial r}\; \end{array}\right).$$

**FIGURE 4:**
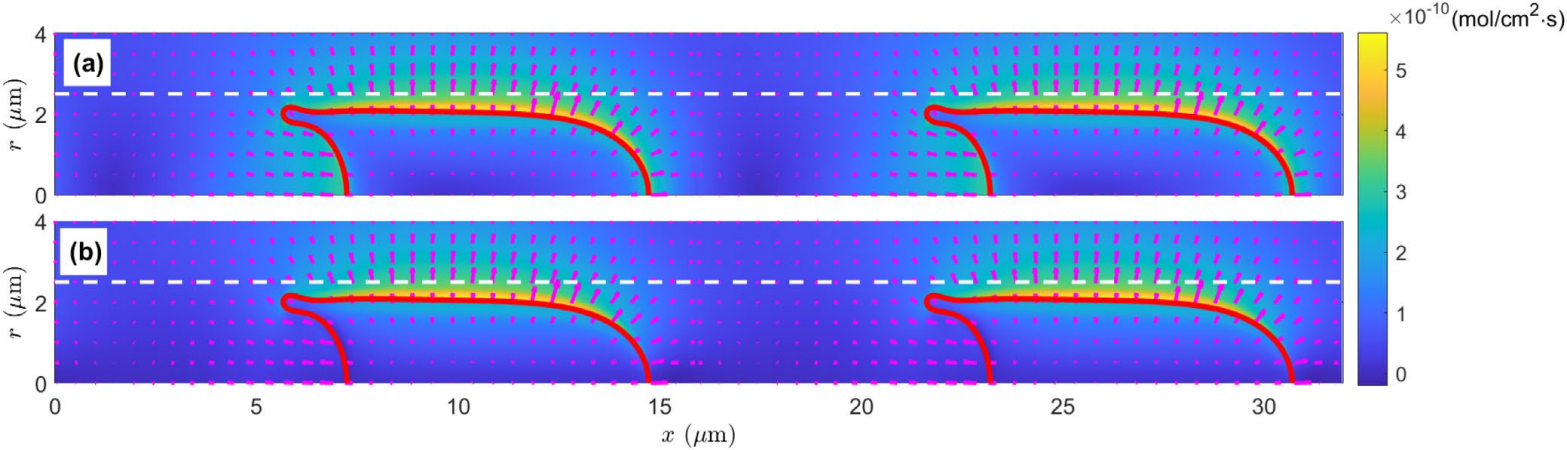
The spatial distribution of the diffusive oxygen flux around RBCs. The oxygen flux is indicated by arrows in both (a) and (b); however, the background color scale represents the magnitude of the overall oxygen flux $\left|j^{d}\right|$ in (a), but it is only for the radial component of the oxygen flux $j_{r}^{d}$ in (b). Panels (a) and (b) share the same color scale for the convenience of direct comparison. The white dashed lines indicate the capillary wall position at r = 2.5 μm, and the deformed RBCs are plotted as solid red curves. Only the first 1.5 μm thickness (r = 2.5–4 μm) of the tissue region is displayed here.

The calculated oxygen flux from this equation is shown in Figure 4a. Unsurprisingly, the strongest oxygen flux occurs near the RBC membrane, especially along the narrow gap between the RBC side surface and the capillary wall. Moreover, considering that the side surface area is proportional to the radial position in a cylindrical system, the contribution of the oxygen flux across the membrane‐wall gap to overall oxygen transport from blood to tissue should be more significant than merely looking at the flux magnitude.

Although the diffusive oxygen flux in Figure 4a exists in all possible directions, depending on the local oxygen gradient, the overall oxygen transport from blood to tissue in the microcirculation is in the radial direction outward. Pure axial flux is not helpful for oxygen transport in the transverse radial direction. For this reason, we replot Figure 4a,b with the background color scale for the radial component $j_{r}^{d}$ only. Comparing Figure 4a,b, one can see that there is not much change in the gap region between the RBC membrane and the capillary wall, since the oxygen flux is approximately in the outward radial direction in that area. However, near the cell's front and rear ends, the oxygen flux is mainly in the axial direction, and the radial part is relatively small. The peak diffusive flux near the RBC membrane in the gap region is ∼ 4 × 10^−10^ mol/cm^2^·s.

**FIGURE 5:**
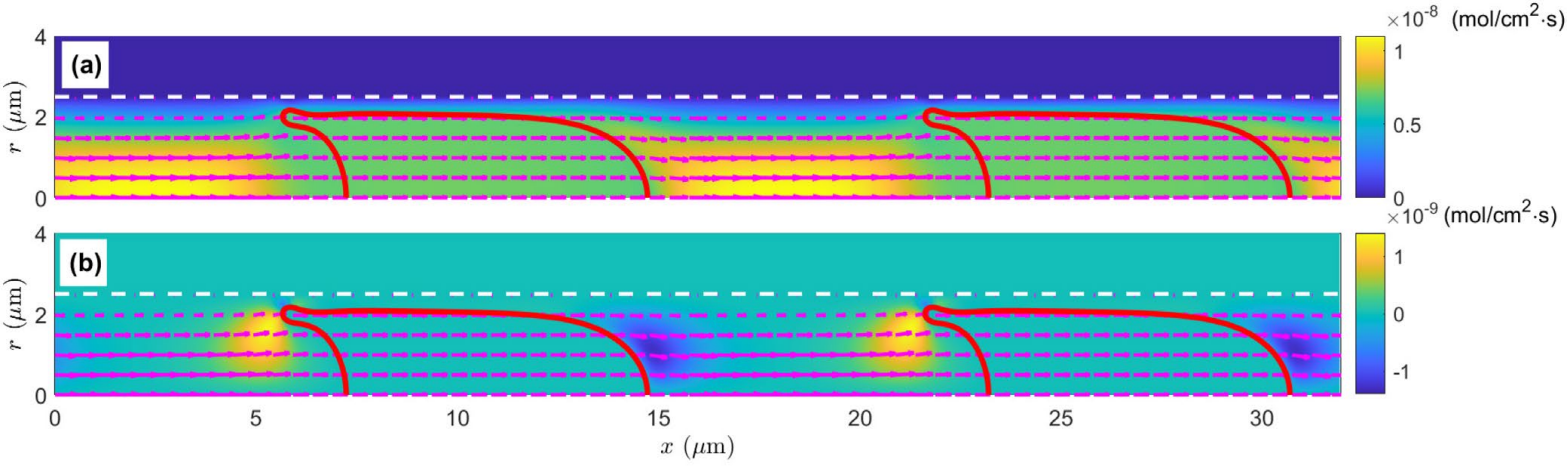
The spatial distribution of the convective oxygen flux around the RBCs. The oxygen flux is indicated by arrows in both (a) and (b); however, the background color scale represents the magnitude of the overall oxygen flux $\left|j^{c}\right|$ in (a), but it is only for the radial component of the oxygen flux $j_{r}^{c}$ in (b). Different color scales are adopted for panels (a) and (b) for the large magnitude difference of the axial and radial flux components. The white dashed lines indicate the capillary wall position at r = 2.5 μm, and the deformed RBCs are plotted as solid red curves. Only the first 1.5 *μ*m thickness (r = 2.5–4 μm) of the tissue region is displayed here.

Another mechanism for mass transfer is the flow convection, and we have the convective oxygen flux due to the plasma flow as

(6) $$j^{c}=\left(\begin{array}{c} j_{x}^{c}\\ j_{r}^{c} \end{array}\right)=\left(\begin{array}{c} u_{x}C_{O_{2}}\;\\ u_{r}C_{O_{2}}\; \end{array}\right),$$

where $u_{x}$ and $u_{r}$ are the axial and radial velocity components, respectively. Here, we use the real velocity shown in Figure 2a instead of the transformed velocity in Figure 2b for the convective flux calculation, and the calculation results are similarly shown in Figure 5. Unlike the diffusive flux in Figure 4a, here in Figure 5a the convective flux, although the magnitude is orders higher (∼ 10^−8^ mol/cm^2^·s), is mainly in the axial direction, and therefore its contribution to the meaningful outward oxygen transport could be nonessential. After the removal of the axial component $j_{x}^{c}$, the radial component $j_{r}^{c}$ in Figure 5b is only large in two small regions, one before and one after the RBC. The magnitude is also significantly reduced to the order of 10^−9^ mol/cm^2^·s. Moreover, the plasma radial current and the convective current in these two regions are of *opposite* directions: inward before the cell and outward behind the cell.

**FIGURE 6:**
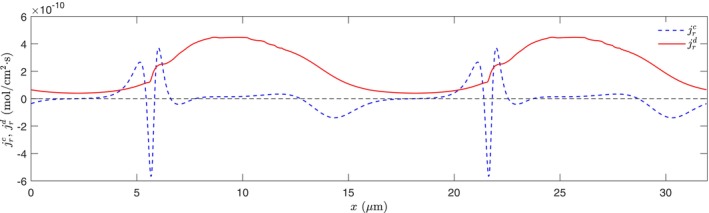
The variation profiles of the radial diffusive ($j_{r}^{d}$) and convective ($j_{c}^{d}$) flux components along the axial direction at the radial position r = 2.25 μm.

Even with a still stronger magnitude, the limited areas and the opposite directions of the radial convective flux $j_{r}^{c}$ may make one wonder how significant it could be to the overall oxygen transport from the blood in the capillary to the tissue. For a more quantitative analysis, in Figure 6, we plot the variation profiles of the radial flux components $j_{r}^{d}$ and $j_{r}^{c}$ at r = 2.25 μm, approximately the hallway position across the narrow membrane‐wall gap. This location is selected for two considerations: it is close to the capillary wall to represent the oxygen flux going into the tissue region; and it is outside of the RBC so the oxygen‐hemoglobin dissociation in the cytoplasm is not involved. We see the diffusive flux is always positive (i.e., going outward to the tissue), and its variation is relatively smooth: from the low value of ∼ 5 × 10^−11^ mol/cm^2^·s in the plasma segment (x = 15–22 μm) to the high flux of ∼ 4 × 10^−10^ mol/cm^2^·s in the membrane‐wall gap (x = 7–13 μm). Meanwhile, the convective flux is negligible except in the small regions before (x = 13–16 μm) and after (x = 4–7 μm) the RBC. The abrupt fluctuation in x = 4–7 μm is associated with the local complex flow field caused by the highly deformed RBC end tip (see Figure 2b). Additionally, the convective flux varies between positive (outward to the tissue) and negative (inward to the capillary centerline) values in these regions, and the net influence on overall oxygen transport could be even less important. For a more direct comparison, we calculate the average values of radial fluxes in Figure 6 and obtain ${\bar{j}}_{r}^{d}$ = 2.2215 × 10^−10^ mol/cm^2^·s and ${\bar{j}}_{r}^{c}$ = 1.6362 × 10^−13^ mol/cm^2^·s the latter is approximately three orders of magnitude smaller. This indicates that the convective flux, although large in some locations, has a negligible net influence on overall oxygen transport.

### 3.3 Turning off Plasma Convection

**FIGURE 7:**
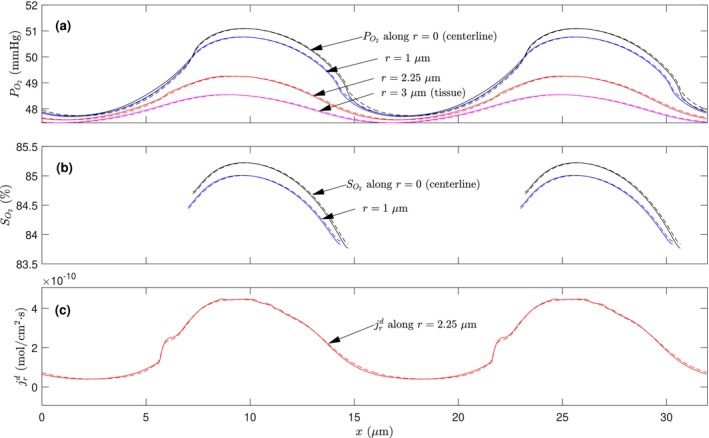
Comparisons of the distribution profiles of $P_{O_{2}}$ (a), $S_{O_{2}}$ (b), and $j_{r}^{d}$ (c) at different radial positions from simulations with the plasma flow around RBCs considered (solid lines) or ignored (dashed lines).

Previous studies had often assumed a negligible effect of plasma convection and neglected detailed plasma flow around the RBC in simulations [16, 17]. In this treatment, a uniform velocity $U_{c}$ is assigned to the entire capillary lumen region (including plasma and RBC). Although we just observed that the convective flux is insignificant when compared to the diffusive counterpart, turning off the plasma flow might affect the oxygen concentration distribution and hence the diffusive flux field as well. To evaluate the effect of plasma flow on oxygen distribution, we re‐do our calculation, however, with the relative flow velocity of plasma around the RBCs being set as zero. Figure 7 compares the axial variations of $P_{O_{2}}$, $S_{O_{2}}$, and $j_{r}^{d}$ at different radial locations from these two sets of calculations. It is evident that all of these variables remain almost unchanged when the relative plasma flow is turned off. The average diffusive flux values at r = 2.25 *μ*m calculated from the $j_{r}^{d}$ profiles in Figure 7c are 2.2215 × 10^−10^ mol/cm^2^·s when the plasma flow is considered versus 2.2185 × 10^−10^ mol/cm^2^·s when it is neglected. The close resemblance between these curves and the nearly identical average values of the diffusive flux indicates that the plasma flow not only contributes very little via direct convective oxygen transfer but also has a negligible influence on the oxygen distribution. This comparison supports the previous treatment of ignoring plasma flow in the literature [16, 17].

### 3.4 On the Peclet Number

In heat and mass transfer, the Peclet number Pe is often used to characterize the relative strength of convective and diffusive contributions [7], and it has been considered in previous studies when discussing the plasma convection effect on oxygen transport [8, 9, 25]. Physically, the Peclet number can be considered as the ratio of the characteristic times ${\tilde{t}}_{d}$ for diffusion and ${\tilde{t}}_{c}$ for convection:

(7) $$\mathrm{Pe}=\frac{{\tilde{t}}_{d}}{{\tilde{t}}_{c}},$$

where

(8) $${\tilde{t}}_{d}=\frac{{\tilde{L}}_{d}^{2}}{D},{\tilde{t}}_{c}=\frac{{\tilde{L}}_{c}}{\tilde{U}}.$$

Here, ${\tilde{L}}_{d}$ and ${\tilde{L}}_{c}$ are the characteristic lengths for the diffusion and convection processes, respectively, and $\tilde{U}$ is the characteristic flow velocity. The diffusivity D should be taken as $D_{O_{2}}$ in plasma. In general practice, the same length scale $\tilde{L}$ is adopted, i.e., ${\tilde{L}}_{c}={\tilde{L}}_{d}=\tilde{L}$, and thus we have

(9) $$\mathrm{Pe}=\frac{\tilde{U}\tilde{L}}{D},$$

which is the classical expression for *Pe*?

For the system we are studying, if we use the capillary radius $R_{c}$ = 2.5 μm for $\tilde{L}$ and the RBC velocity $U_{c}$ = 1 mm/s for $\tilde{U}$, the resulting Peclet number is 1.042, suggesting that the plasma convection is of similar or comparable importance as the mass diffusion for oxygen transport. Obviously, this does not agree with our simulation results presented above: the plasma convection effect is orders weaker than the diffusion effect. To clarify this confusion, we look at the $P_{O_{2}}$ and diffusive flux distributions in Figures 3 and 4. Apparently, the oxygen diffusion is most profound across the membrane‐wall gap, and therefore, the gap width (∼ 0.4 *μ*m) is more appropriate for the diffusion characteristic length ${\tilde{L}}_{d}$. Moreover, in our system, the interesting oxygen transport is along the radial direction, and then the radial component of plasma velocity, which has a maximum value of 0.163 mm/s in our system, is more meaningful for the characteristic velocity $\tilde{U}$. Using these revised values, we now recalculate the Peclet number $Pe^{\prime }$ for our specific system as

(10) $$\mathrm{Pe}=\frac{{\tilde{t}}_{d}}{{\tilde{t}}_{c}}=\frac{{\left(0.4\times 10^{-6}\;m\right)}^{2}/\left(2.4\times 10^{-9}\;m^{2}/s\right)}{\left(2.5\times 10^{-6}\;m\right)/\left(0.163\times 10^{-3}\;m/s\right)}=4.35\times 10^{-3}.$$

This small value (∼ 10^−3^) of the modified Peclet number agrees with our observation of the weak convection effect in previous sections.

## 4 Summary and Conclusions

In this research, we combined the boundary integral and lattice Boltzmann methods to simulate the RBC deformation, plasma flow, and oxygen transport in a capillary. Apart from previous studies, we calculated the oxygen fluxes from concentration gradient‐induced diffusion and plasma circulation‐induced convection. Our results reveal that, although of a much larger magnitude, the convective flux only has a negligible influence on the overall oxygen transport from the capillary blood to tissues, while the diffusive flux plays a determinant role. Ignoring the plasma convection in gas transport simulations is acceptable since this treatment only affects the results very slightly. We also re‐examined the definition of the Peclet number and proposed to use different characteristic lengths for the diffusion and convection processes for capillary blood flows. The Peclet number value of our revised definition is orders of magnitude smaller than that of the classical expression, and it is more appropriate and meaningful for the specific situation of microcirculatory gas transport. The study could be helpful in clarifying long‐standing concerns about the effect of plasma convection on gas transport in the microcirculation.

## 5 Perspectives

The results and analysis presented in this paper are based on simulations of oxygen transport using a specific set of parameters; however, we believe that the general findings could be extended to other relevant situations. For example, the blood velocity in capillaries could be a few times higher than the 1 mm/s cell velocity we used here; however, since the convective contribution is only approximately one‐thousandth of that from diffusion in our results, the plasma convection would still be negligible in systems with a higher blood velocity. Another important parameter is the hematocrit, which will change RBC separation distance *L* but has a relatively minor effect on cell deformation. Therefore, a higher or lower hematocrit will mainly change the length of the plasma circulation eddy, but not much for the flow structure around the RBC front and rear ends. The convective flux features observed in this study, including the limited areas where the radial flux is significant and the opposite directions in these areas, will still be true. Furthermore, although oxygen transport is considered in this study, the information revealed could also be valid for the transport processes of gas species such as CO and nitric oxide (NO) in the microcirculation.

## Conflicts of Interest

The author declares no conflicts of interest.

## Data Availability

The author declares that the data supporting the findings of this study are available in the article.
